# Association between Egg Consumption and Cholesterol Concentration: A Systematic Review and Meta-Analysis of Randomized Controlled Trials

**DOI:** 10.3390/nu12071995

**Published:** 2020-07-04

**Authors:** Man-Yun Li, Jin-Hua Chen, Chiehfeng Chen, Yi-No Kang

**Affiliations:** 1 School of Medicine, College of Medicine, Taipei Medical University, Taipei 110, Taiwan; b101106112@tmu.edu.tw; 2 Graduate Institute of Data Science, College of Management, Taipei Medical University, Taipei 110, Taiwan; jh_chen@tmu.edu.tw; 3 Research Center of Biostatistics Center, College of Management, Taipei Medical University, Taipei 110, Taiwan; 4 Biostatistics Center, Wan Fang Hospital, Taipei Medical University, Taipei 116, Taiwan; 5 Department of Public Health, School of Medicine, College of Medicine, Taipei Medical University, Taipei 110, Taiwan; 6 Division of Plastic Surgery, Department of Surgery, Wan Fang Hospital, Taipei Medical University, Taipei 116, Taiwan; 7 Cochrane Taiwan, Taipei Medical University, Taipei 110, Taiwan; 8 Evidence-Based Medicine Center, Wan Fang Hospital, Taipei Medical University, Taipei 116, Taiwan; 9 Research center of big data and meta-analysis, Wan Fang Hospital, Taipei Medical University, Taipei 116, Taiwan; 10 Institute of Health Behaviors and Community Sciences, College of Public Health, National Taiwan University, Taipei 106, Taiwan

**Keywords:** egg consumption, low-density lipoprotein, high-density lipoprotein

## Abstract

The association of egg consumption and serum cholesterol concentrations in healthy people has been discussed for a long time. In this study, we aimed to explore association of egg consumption with on low-density lipoprotein cholesterol (LDL-c) and high-density lipoprotein cholesterol (HDL-c) concentrations and the LDL-c/HDL-c ratio through meta-analysis. This systematic review only included randomized controlled trials (RCTs) investigating egg consumption in healthy populations without combination therapy. We extracted mean and standard deviation for LDL-c/HDL-c ratio, LDL-c/HDL-c. The extracted data were pooled in a random-effects model and were presented as mean difference (MD) with 95% confidence interval (CI). Moreover, subgroup analyses were conducted for understanding effects of more egg consumption (MEC) on different intervention periods, egg-consumption levels, classification of responders. Overall, 17 RCTs met the eligibility criteria and pooled results showed MEC group had a higher LDL-c/HDL-c ratio than the control group (MD = 0.14, *p* = 0.001, I^2^ = 25%). The MEC group also had higher LDL-c than the control group (MD = 8.14, *p* < 0.0001, I^2^ = 18%). Moreover, for the subset of intervention over two months, the MEC group seemed to have a larger effect size than the subset of intervention within two months. This synthesis, the largest meta-analysis on this topic, shows the impact of egg consumption on lipid profiles among healthy subjects. Notably, longer time with MEC may lead to higher LDL-c/HDL-c ratio and LDL-c. However, RCTs with long tern follow-up are needed to guarantee the association between egg consumption and human health.

## 1. Introduction

The American Heart Association recommends that total dietary cholesterol consumption should be <300 mg/day [1]. A large egg yolk contains approximately 275 mg of cholesterol—near the recommended daily limit of cholesterol intake [2]. The 2015–2020 Dietary Guidelines for Americans removed the limitation of dietary egg consumption [3,4]. One study indicated that dietary cholesterol contained in whole eggs is not well-absorbed and is not associated with longer-term plasma cholesterol control [5], while other studies indicated that the concentration of low-density lipoprotein cholesterol (LDL-c) and high-density lipoprotein cholesterol (HDL-c) significantly increased in an egg-consuming group [6,7]. To clarify this controversy, some systematic reviews tried to determine whether egg consumption influences cholesterol levels, but most of them only focused on LDL-c, HDL-c, total cholesterol and triglycerides [8,9,10,11]. However, current evidence indicates that LDL-c/HDL-c ratio is a better predictor of cardiovascular diseases than isolated parameters [12]. To our best knowledge, only one of the previous systematic reviews investigated LDL-c/HDL-c ratio, and concluded that egg consumption had insignificant effect on LDL-c/HDL-c ratio [9]. Unfortunately, the conclusion was not based on all of the available data. There is still a need to reexamine this issue using a larger scale.

Eggs are an important food source because they contain many proteins, lipids and minerals [13]. A boiled egg (50 g) provides 6.29 g protein, 0.56 g carbohydrate, 1.6 g saturated fat, 2.0 g monounsaturated fat and 0.7 g polyunsaturated fat. Moreover, eggs contain various minerals (calcium, iron, magnesium and phosphorus) and many vitamins except vitamin C [14]. After consuming eggs, the intestines are responsible for absorbing dietary cholesterol. Cholesterol absorption includes dietary cholesterol and biliary cholesterol. Between dietary and biliary cholesterol, biliary cholesterol accounts for most of the cholesterol in intestine. Biliary cholesterol is about two grams/day while dietary cholesterol is 0.4 g/day according to the average American diet. The absorption rate varies in population. The average rate is approximately 50% [15]. After absorption, free cholesterol is esterified by enterocytes by action of acyl-coenzyme A cholesterol acyl-transferase [16]. Then, it is transported inside chylomicrons into the peripheral circulation [17]. Next, chylomicrons form chylomicron remnants under the action of lipoprotein lipase. The uptake of chylomicron remnants is mediated by the LDL receptor, which recognizes apolipoprotein B 100 (Apo B 100) and apolipoprotein E (Apo E). Through endocytosis, chylomicrons can be taken up in liver and most other tissues [18,19].

Hyperlipidemia is a phenomenon of increased levels of lipids or triglycerides [20]. It can be classified as hypercholesterolemia, hypertriglyceridemia and elevation of both cholesterol and triglycerides [21]. Higher amounts of LDL-c result in higher risks of cardiovascular diseases. There are more than 100 million people in the United States with elevated LDL-c concentrations, so they have higher risks of cardiovascular diseases [22]. The global prevalence of elevated total cholesterol among adults are 39% [23]. The intake of dietary cholesterol is associated with increased LDL-c concentration [11]. However, this is an oversimplification since the response of serum cholesterol to dietary cholesterol intake is quite complicated. Humans can endogenously produce cholesterol, and most of the cholesterol in the body comes from biosynthesis [24]. Due to this controversy, several studies have examined the relationship between egg consumption and cholesterol levels. Some used different doses of egg consumption while others combined different diets [25,26].

In the previous systematic review, they provided six randomized controlled trials to analyze the effect of LDL-c/HDL-c ratio while we used more RCTs to strengthen the evidence [9]. Due to incomplete information, we conducted a systematic review to examine the relationship between egg consumption and lipid profiles, especially the association of egg consumption with LDL-c/HDL-c ratio. Through this study, we would like to foster a better understanding of this controversy.

## 2. Methods

This comprehensive review was conducted according to the Cochrane handbook, and the systematic review and meta-analysis were according to PRISMA guidelines [27]. Patient consent was not required as this study used published data; therefore, it was also exempted from institutional review board approval. The protocol was registered in PROSPERO (CRD42019138623).

### 2.1. Eligibility Criteria, Evidence Search and Study Selection

We only included randomized clinical trials (RCTs), while excluding case control studies and cohort studies. Eligible trials only investigated healthy subjects; we excluded those trials that only recruited patients with hypercholesterolemia, diabetes or hypertension because lipid levels would be higher in patients with hypertension [28]. There was no limitation on the age of participants in eligible trials. The intervention for the experimental group was consumption of eggs without accompanying buttermilk drinks, because a previous study showed that consumption of buttermilk and skimmed milk influences the effects of egg yolks on lipoprotein [29]. In addition, we excluded studies that used palm oil in the diet because it has a hypercholesterolemic effect [30]. The control group in the previous literature usually refers to regular egg consumption, a high-carbohydrate diet or the same amount of an egg substitute. Regular egg consumption represented regular diet of every individual. Moreover, there was no limitation on the method of cooking the eggs.

We screened the PubMed and EMBASE databases before May 2020. The primary search strategy was (cholesterol OR lipoprotein OR LDL-c OR HDL-c) AND (egg OR eggs OR yolk OR yolks) with a restriction of RCT. The search strategy had no publication date limitation.

After relevant references were identified from online databases, one investigator independently did initial screening for eligible references according to the criteria, and another one double-checked for the appropriateness of evidence selection. Then, we had a meeting to confirm eligible trials. A third investigator participated in the meeting for making the final judgment of evidence selection.

### 2.2. Data Extraction

Baseline and outcome data were extracted. We extracted information on age, gender, intervention time, the amount of egg consumption and outcomes. Moreover, we also extracted information on absorption hypo-responders and hyper-responders to cholesterol. Because outcomes were continuous, we extracted the mean and standard deviation (SD) for the meta-analysis.

As continuous outcomes were presented in different scales, we used a lipid conversion factor (1 mmol/L = 38.67 mg/dL [31]) to convert mmol/L to mg/dL and used the mean difference (MD) and 95% confidence interval (CI) in forest plots. When the value of the LDL-c/HDL-c ratio was not available in the original report, we use Taylor approximation to estimate its value: E(x/y) ≈ {E(x)/E(y) × (1+CVy×(CVy − Corr (x,y) × CVx))} and V(x/y) ≈ ((E(x))^2^/(E(y))^2^)(σ^2^x/(E(x))^2^ − 2 × CV(x,y)/E(x) E(y) + σ^2^y/(E(y))^2^) [32,33]; where x represents LDL-c and y represents HDL-c. E(x/y) is an exceptional value of LDL-c/HDL-c. E(x) and E(y) populations are respective concentrations of LDL-c and HDL-c and we estimated them from our collected studies. Corr(x,y) is the correlation coefficient between LDL-c and HDL-c and we estimated the value to be 0.195 according to Weggemans et al. [11]. CVx and CVy are coefficients of variation of LDL-c and HDL-c, respectively, and we estimated them from our collected studies. σ^2^x represents the variance of LDL-c, while σ^2^y represents the variance of HDL-c. CV(x,y) is the covariance of LDL-c and HDL-c.

### 2.3. Quality Assessment

The quality of the included trials was determined using the quality assessment tool for quantitative studies, which was developed by the effective public health practice project [34]. There are six components in the quality assessment tool: selection bias, study design, confounders, blinding, data collection methods, withdrawals and dropouts. Each component was ranked as strong, moderate or weak according to the guidelines of the quality assessment tool for quantitative studies [34].

### 2.4. Evidence Synthesis and Statistical Analysis

We used Review Manager 5.3 (The Nordic Cochrane Centre, The Cochrane Collaboration, Copenhagen, Denmark) to analyze our extracted data and estimated pooled results in a random-effects model. Because our outcomes were continuous data, we presented MDs and 95% CIs for pooled estimates. We also did subgroup analysis according to the intervention times and the amount of eggs consumed. Because the treatment of experimental groups varied widely, we subdivided experimental groups into additional 1 egg, 1, 2 and ≥ 3 eggs/day according to egg-consumption levels. All the experimental group are called MEC group. Intervention times were divided into ≤ 2 months and > 2 months. Then, we used I^2^ statistic to measure heterogeneity. A rough guide for heterogeneity is that > 50% may indicate substantial heterogeneity [35]. We further conducted a sensitivity analysis to confirm that the pooled results were not influenced by individual studies. Publication bias was assessed by a funnel plot and Egger’s test.

## 3. Results

**Figure 1:**
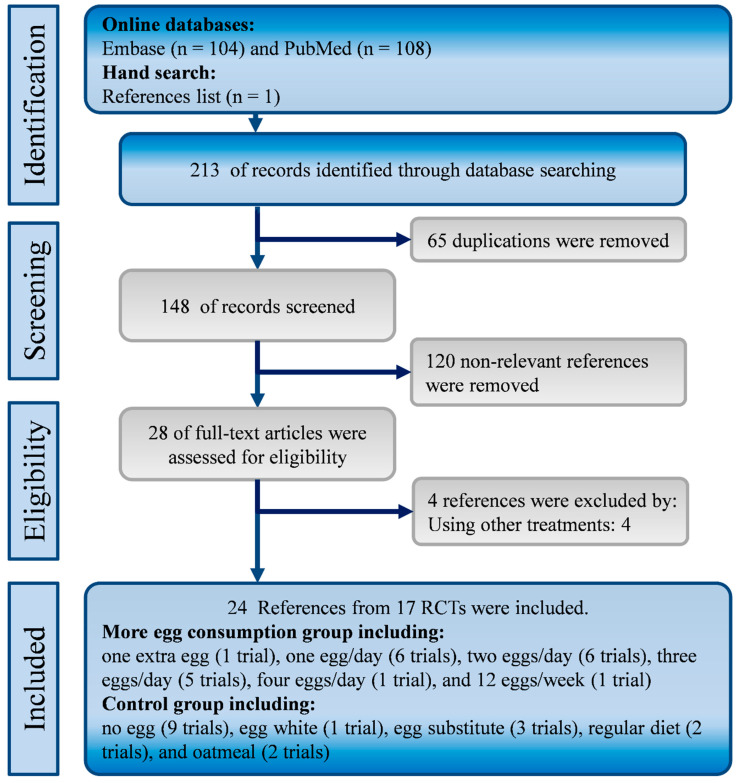
Flowchart of the systematic review and meta-analysis according to PRISMA guidelines.

Our initial search included 213 studies from PubMed, EMBASE and hand searching. After duplicates were removed, 148 references were screened by title and abstract and we excluded 120 non-relevant references. The full text of several remaining references was excluded because of using other treatments (*n* = 4). Therefore, we included 24 references from 17 RCTs [6,7,36,37,38,39,40,41,42,43,44,45,46,47,48,49,50,51,52,53,54,55,56,57]. A flowchart shows the screening and selection processes (Figure 1).

### 3.1. Characteristics and Quality of Included Studies

We included 17 randomized clinical trials [7,36,40,41,42,43,44,45,46,47,48,51,53,54,55,56,57]. Table below shows an overview of the characteristics of the included studies. All subjects in the studies we included were healthy. Studies with subjects that had hypertension, diabetes or hypercholesterolemia were excluded. Six studies were RCTs [40,42,47,53,54,56]. Eleven studies were crossover studies [7,36,41,43,44,45,46,48,51,55,57]. The intervention time ranged 21–84 days (Table 1). The quality of all studies was acceptable (Table S1).

### 3.2. LDL-c/HDL-c Ratio

**Figure 2:**
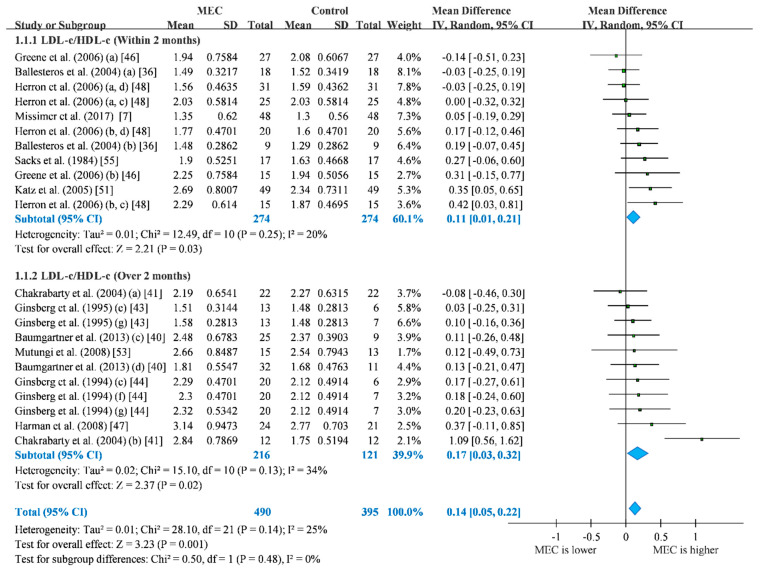
Forest plot of LDL-c/HDL-c ratio. For studies that the lipid levels were reported separately, we presented in different rows in forest plots. (a) hypo-responder group; (b) hyper-responder group; (c) men group; (d) women group; (e) one-egg group; (f) two-eggs group; (g) ≥ three-eggs group; blue: *p* < 0.05. Blue refers to statistical significance in pooled analysis.

Our primary outcome was the LDL-c/HDL-c ratio and relevant data were available from 12 trials (Figure 2). We excluded data from the trial by Rueda et al. [54] because the data had different included participants between measurements of LDL-c and HDL-c. Therefore, we could not estimate the LDL-c/HDL-c ratio by Taylor’s approximation [32,33]. Overall, more egg consumption (MEC) group exhibited significant elevation in the ratio than did control groups (MD = 0.14; 95% CI: 0.05 to 0.22; I^2^ = 25%). Based on the intervention time, we divided the included studies into subgroups of within 2 months and over 2 months. The MEC group presented significant higher LDL-c/HDL-c ratio than control group in both subgroups of intervention within 2 months (MD = 0.11; 95% CI: 0.01 to 0.21; I^2^ = 20%) and over 2 months (MD = 0.17; 95% CI: 0.03 to 0.32; I^2^ = 34%). The result of LDL-c/HDL-c ratio may not be affected by any single study (Figure S1) and publication bias (Figure S2).

### 3.3. Low-Density Lipoprotein Cholesterol

**Figure 3:**
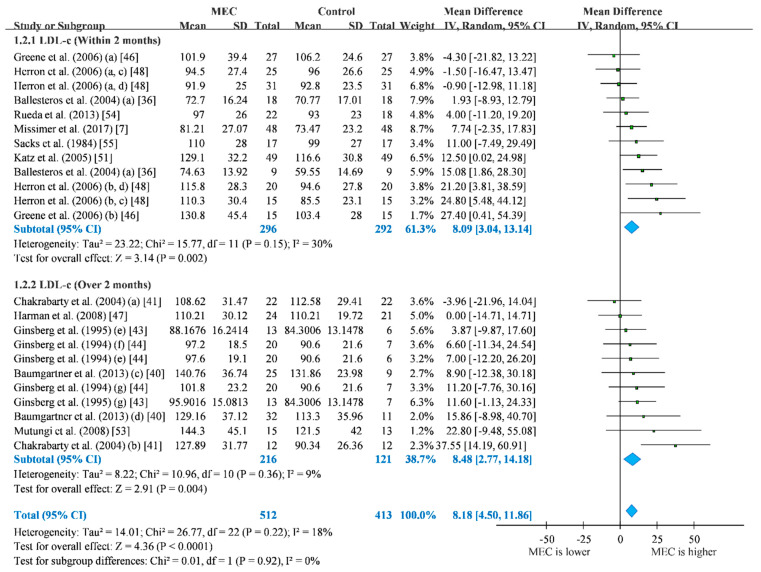
Forest plot of low-density lipoprotein cholesterol (mg/dL). For studies that the lipid levels were reported separately, we presented in different rows in forest plots. (a) hypo-responder group; (b) hyper-responder group; (c) men group; (d) women group; (e) one-egg group; (f) two-eggs group; (g) ≥ three-eggs group; blue: *p* < 0.05. Blue refers to statistical significance in pooled analysis.

The 13 RCTs we included in this study showed that the MEC group had a significantly higher concentration of LDL-c than the control group (MD = 8.14; 95% CI: 4.46 to 11.82; I^2^ = 18%) Based on the intervention time, we discovered that the MEC group exhibited higher LDL-c than the control group in both subgroups of intervention within 2 months (MD = 8.09 mg/dL; 95% CI: 3.04 to 13.14, I^2^ = 30%) and over 2 months (MD = 8.48 mg/dL; 95% CI: 2.77 to 14.18, I^2^ = 9%). Longer intervention period may lead to greater LDL-c concentration (Figure 3). The result of LDL-c may not be affected by any single study (Figure S3) and publication bias (Figure S4).

### 3.4. High-Density Lipoprotein Cholesterol

**Figure 4:**
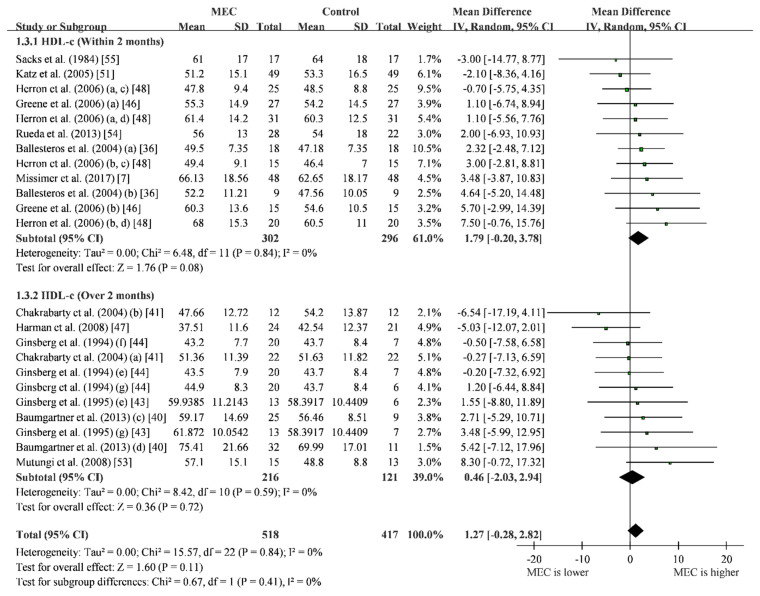
Forest plot of high-density lipoprotein cholesterol (mg/dL) for studies that the lipid levels were reported separately, we presented in different rows in forest plots. (a) hypo-responder group; (b) hyper-responder group; (c) men group; (d) women group; (e) one-egg group; (f) two-eggs group; (g) ≥ three-eggs group. Black diamond refers to no statistical significance in pooled analysis.

We divided 13 RCTs into two groups according to the intervention time. Based on the included studies, the MEC group did not show significant difference with the control group (MD = 1.27; 95% CI: −0.28 to 2.83; I^2^ = 0%) when pooled together. Similar results also showed in the subgroup by intervention duration (Figure 4). The result of HDL-c may not be affected by any single study (Figure S5) and publication bias (Figure S6).

### 3.5. Further Analysis

Concerning egg-consumption level, we divided studies into three groups: one egg, two eggs and three or more eggs (Table 2). With one-egg-consumption and three-or-more-eggs-consumption, the MEC group did not have significant difference from control group, while MEC group of two-eggs-consumption showed significantly higher LDL-c/HDL-c ratio (MD = 0.13; 95% CI: 0.01 to 0.26; I^2^ = 13%). Interestingly, we found that two included studies [46,48] in the three-or-more-eggs subgroup had a shorter intervention time of within 2 months, which accounted for 53.7% of participants. Therefore, length of intervention period may be the main reason for the increased LDL-c/HDL-c ratio.

In the subgroup analysis for LDL-c, one-egg group (MD = 8.37; 95% CI: 1.06 to 15.69; I^2^ = 22%), two-egg group (MD = 7.32; 95% CI: 2.20 to 12.44; I^2^ = 0%) and three-or-more-eggs group (MD = 9.87; 95% CI: 2.09 to 17.65; I^2^ = 42%) resulted in significantly higher LDL-c than control group.

About the subgroup analysis for HDL-c, one-egg (MD = 0.28; 95% CI: −2.85 to 3.42; I^2^ = 0%) and two-eggs (MD = 0.35; 95% CI: −2.35 to 3.04; I^2^ = 0%) showed no significant lower elevations than control group. Interestingly, in the subgroup of three-or-more-eggs, MEC had higher HDL-c than control group (MD = 2.55 mg/dL; 95% CI: 0.16 to 4.94; I^2^ = 0%). Therefore, egg consumption only slightly influenced the HDL-c.

## 4. Discussion

### 4.1. Summary of Main Results

Our study provides strong evidence on the topic of the impact of egg consumption on lipid profiles through synthesizing more than 10 trials. Compared to Rouhani et al. [9], we included studies that were published before 2000. The results indicated that egg consumption significantly increases the LDL-c/HDL-c ratio and LDL-c levels, especially with a longer intervention duration. Yet, egg consumption did not effectively increase the HDL-c level. Our pooled results revealed some significant findings among subgroups of egg-consumption levels, but the results did not show a clear trend of increment with the egg-consumption level.

### 4.2. Overall, Completeness and Applicability of the Evidence

As to the primary outcome, pooled results of the LDL-c/HDL-c ratio, we found significantly higher value in the MEC group than in the control group (MD = 0.14). For the primary prevention of cardiovascular diseases, the target value of LDL-c/HDL-c for men is < 3.0, while for women it is < 2.5. The risk level for men is > 3.5, while for women it is > 3.0 [12]. Moreover, LDL-c/HDL-c ratio is associated with increased risk of sudden cardiac death and cardiovascular diseases, which includes coronary artery disease, cerebrovascular disease, peripheral artery disease and aortic atherosclerosis [12,58,59]. Thus, those with a borderline LDL-c/HDL-c ratio should restrict their egg consumption because higher egg consumption increased LDL-c/HDL-c ratio by 0.14. According to our evidence, we found potential reasoning for this phenomenon from the pooled estimates of differences in LDL-c and HDL-c concentrations. Results showed that egg consumption did not increase HDL-c, but increased LDL-c. Therefore, egg consumption affects the LDL-c/HDL-c ratio.

HDL-c plays an important role in reverse cholesterol transport. HDL-c takes cholesterol from peripheral tissue and transports to liver for excretion into the feces [60]. For HDL-c, our evidence only showed a very little effect size with non-significance, especially in the subgroup with over two months of treatment. LDL-c is a fat that circulates in vessels, moving to the tissue that is needed for cell repair. It also accumulates inside of arterial wall [19]. The target value of LDL-c is less than 100 mg/dL. When the concentration reaches 130 mg/dL to 159 mg/dL, it means borderline high [61]. For LDL-c, a non-extreme short-term intervention (16 weeks) of daily cholesterol consumption led to increased LDL-c. This result was consistent with another study [30]. Thus, people with borderline high LDL-c level should restrict their egg consumption. A further study revealed that MEC resulted in increased LDL-c and HDL-c concentrations more than oatmeal breakfast and breakfast [52]. Egg white had an effect of decreasing total cholesterol and increasing HDL-c compared to tofu and cheese [62]. Moreover, different cooking methods resulted in different amounts of cholesterol. For example, there are three common types of egg products in China. All of them are cooked eggs, but with different amounts of cholesterol per 100 g [63]. As to egg consumption with additional food or drinks, one of the included studies indicated that consuming eggs with buttermilk caused increases in LDL-c and total cholesterol to become statistically insignificant [40]. When consuming choline bitartrate supplement as control group, the study revealed that LDL-c/HDL-c ratio did not show significant elevation in MEC group while our results showed a slight elevation in LDL-c/HDL-c ratio with significance. Therefore, we consider choline bitartrate supplement to be a factor that slightly increase LDL-c/HDL-c ratio, making the elevation of LDL-c/HDL-c ratio in MEC group become insignificance [26]. When it comes to different amount of egg consumption, our study cannot determine dose effect. Most of our findings echo to a study by Dimarco et al., in which the effects of different amount of egg consumption on cholesterol level were evaluated [25]. Increasing dose of egg intake seems to associate with higher HDL-c level. However, it is still unclear in association of increasing dose of egg consumption and LDL-c or LDL-c/HDL-c ratio. On the basis of previous synthesis, we also notice that the results of LDL-c and LDL-c /HDL-c ratio may be driven by responders [9]. However, random-effects model was applied for all of our analyses. In the statistical model, variances of samples among trials are taken in to consideration; and thus, the results are close to the effects in the entire population in the synthesis. The results may not be seriously biased by the responders.

### 4.3. Comparison to Previous Syntheses

Before our synthesis, there were three important meta-analyses on the topic of egg consumption and lipoprotein levels [9,10,11]. Most meta-analyses indicated that egg consumption will increase LDL-c and HDL-c [9,10,11]. An earlier one in 2001 used limited studies and mainly analyzed LDL-c and HDL-c [11]. The other two previous syntheses had insufficient evidence for identifying the impact of egg consumption on the LDL-c/HDL-c ratio, although they found significant findings in separate pooled LDL-c and HDL-c concentrations [9,10]. One of the recent syntheses included 27 references on this topic, but only pooled seven of them from six RCTs in the meta-analysis of LDL-c/HDL-c ratio. Then, they found highly heterogenous estimate even in some subgroup analysis [9]. Another one synthesis by Wang et al. in 2019 focused on middle-age and older population and analyzed LDL-c/HDL-c ratio only two RCTs [10]. Those syntheses noted the importance of the LDL-c/HDL-c ratio with some significant findings in some subgroup analyses, while they were prevented from making a perfect quantitative synthesis because of statistical techniques. Our study overcame the statistical barrier of estimating the LDL-c/HDL-c ratio according to a relevant formula [32,33]. That is to say, our evidence provides more-powerful results for understanding the impact of egg consumption on the LDL-c/HDL-c ratio.

In the previous syntheses, moreover, they mixed health subjects and patients. On the contrary, our study tried to avoid bias from conceptual heterogeneity by focusing on healthy subjects. For instance, the best synthesis in recent years included trials by Bautista et al. [30] and Schwab et al. [64], but we did not include those trials because they recruited patients with diabetes or hypercholesterolemia [9]. Thus, pooled results in the present study may be more precise and our findings may be more appropriate for healthy population. Furthermore, we carefully reviewed all trials to avoid overestimating. For example, in a previous meta-analysis, the synthesis double-counted results from the same trial by the study team of Herron et al. [38,39,48,49,50]. Similarly, a trial by the study team of Greene et al. was also double-counted [6,46,57]. The double-counted results may have resulted in overestimates and inaccuracies. That is why our pooled results of HDL-c differed from previous syntheses because of the differences in conceptual heterogeneities and data sources between our synthesis and previous meta-analyses as described in this paragraph.

### 4.4. Limitations

There were some limitations to this study because of potential biases in the synthesis. The intervention duration of the included studies were all within a year. We could not conclude results for longer intervention times. Next, we could not determine the foods accompanying the eggs in every included study. Different background diets may have influenced the results. However, we excluded the intervention that may influence the cholesterol level, we could not tell whether there is other factors due to insufficient information [26]. Then, due to the variation between included studies, MEC group have different amount of egg consumption. However, we used subgroup analysis to explain the result, ≥ three-egg group still had variation. Finally, the LDL-c/HDL-c ratio was the main estimation used to assess the risk of cardiovascular diseases [12]. Not every included study reported the LDL-c/HDL-c value. Thus, we had to estimate the LDL-c/HDL-c value in several studies.

## 5. Conclusions

Based on available evidence, this is the largest meta-analysis in exploring the impact of egg consumption on LDL-c/HDL-c ratio among healthy subjects and reveals that more eggs consumed per day may influence cardiovascular disease risks by increasing LDL-c and the LDL-c/HDL-c ratio. Notably, longer-term high egg-consumption may lead to higher LDL-c/HDL-c ratio and LDL-c. However, RCTs with long tern follow-up are needed to guarantee the association between egg consumption and human health.

## Figures and Tables

**Table 1:** Characteristics of patients in the included studies.

Trial	Male/Female	Age Range	Intervention		Duration	Result (↑: increased concentration; ↓: decreased concentration)
			Egg Group	Control		
Missimer et al. (2017) [7]	24/26	18–30	2 eggs/day	Oatmeal	28 days	LDL-c↑; HDL-c↑
Baumgartner et al. (2013) [40]	34/63	18–65	One extra egg	Regular diet	84 days	TC↑; LDL-c↑
Rueda et al. (2013) [54]	27/46	17–20	1 egg/day	No egg	28 days	Statistically insignificant change
Harman et al. (2008) [47]	14/31	18–55	2 eggs/day	No egg	84 days	Statistically insignificant change
Mutungi et al. (2008) [53]	28/0	40–70	3 eggs/day	No egg	84 days	HDL-c↑
Wal et al. (2008) [56]	10/63	25–60	2 eggs/day	Regular diet	56 days	Statistically insignificant change
Waters et al. (2007) [57]	0/22	50–77	3 eggs/day	No egg	30 days	TC↑; HDL-c↑; LDL-c↑
Herron et al. (2006) [48]	40/51	21–43	3 eggs/day	Egg substitute	30 days	TC↑; HDL-c↑; LDL-c↑
Greene et al. (2006) [46]	13/29	50–80	3 eggs/day	Egg substitute	30 days	HDL-c↑; LDL-c↑
Goodrow et al. (2006 [45])	7/26	60–96	1 egg/day	Egg substitute	35 days	Statistically insignificant change
Katz et al. (2005) [51]	30/19	36–73	2 eggs/day	Oatmeal	42 days	Statistically insignificant change
Ballesteros et al. (2004) [36]	25/29	8–12	2 eggs/day	Egg white	30 days	LDL-c↑; HDL-c↑
Chakrabarty et al. (2004) [41]	22/12	19–32	1 egg/day	No egg	56 days	TC↑; LDL-c↑; TC/HDL-c↑
Ginsberg et al. (1995) [43]	0/13	22–31	3 eggs/day1 egg/day	No egg	56 days	TC↑; HDL-c↑; LDL-c↑
Ginsberg et al. (1994) [44]	24/0	22–31	1 egg/day2 eggs/day4 eggs/day	No egg	56 days	TC↑; LDL-c↑
Garwin et al. (1992) [42]	42/56	41–48	12 eggs/week	No egg	42 days	TC↓; HDL-c↓; LDL-c↓
Sacks et al. (1984) [55]	4/13	18–24	1 egg/day	No egg	21 days	LDL-c↑

**Table 2:** Subgroup analysis.

Outcome/					
Subgroup	Studies	Patients	MD	95% CI	I-Square
LDL-c/HDL-c ratio					
1 egg	5	226	0.20	−0.01–0.42	59%
2 eggs	5	320	0.13	0.01–0.26	13%
≥ 3 eggs	5	339	0.09	−0.02–0.20	0%
LDL-c					
1 egg	6	266	8.37	1.06–15.69	22%
2 eggs	5	320	7.32	2.20–12.44	0%
≥ 3 eggs	5	339	9.87	2.09–17.65	42%
HDL-c					
1 egg	6	276	0.28	−2.85–3.42	0%
2 eggs	5	320	0.35	−2.35–3.04	0%
≥ 3 eggs	5	339	2.55	0.16–4.94	0%

## Supplementary Materials

The following are available online at https://www.mdpi.com/2072-6643/12/7/1995/s1. Table S1: Risk of bias; Figure S1: Sensitivity analysis of LDL-c/HDL-c ratio; Figure S2: Small study effects in LDL-c/HDL-c ratio; Figure S3: Sensitivity analysis of LDL-c; Figure S4: Small study effects in LDL-c; Figure S5: Sensitivity analysis of LDL-c; Figure S6: Small study effects in LDL-c.

## Abbreviations

CI	Confidence interval
HDL-c	high-density lipoprotein cholesterol
LDL-c	low-density lipoprotein cholesterol
MEC	more egg consumption
MD	mean difference
NSR	nonspecific responders
PRISMA	preferred reporting items for systematic reviews and meta-analyses
RCT	randomized clinical trial
SD	standard deviation
