# Supplementary material for: Association between Egg Consumption and Cholesterol Concentration: A Systematic Review and Meta-Analysis of Randomized Controlled Trials
Source: Nutrients. 2020 Jul 4;12(7):1995. doi: 10.3390/nu12071995 (PMC7400894; doi:10.3390/nu12071995)
Supplement: Supplementary file 1 [file nutrients-12-01995-s001.pdf]

Association between egg consumption and cholesterol concentration:

A systematic review and meta-Analysis of randomized controlled trials

(Supplementary Figures)

Figure S1. Sensitivity analysis of LDL-c/HDL-c ratio

Figure S2. Small study effects in LDL-c/HDL-c ratio

Figure S3. Sensitivity analysis of LDL-C

Figure S4. Small study effects in LDL-C

Figure S5. Sensitivity analysis of LDH-C

Figure S6. Small study effects in LDH-C

Figure S1

Sensitivity analysis of LDL/HDL ratio

Study name	Statistics with study removed			
	Point	Lower limit	Upper limit	p-Value
Chakrabarty 2004 (6)	0.105	0.035	0.176	0.003
Katz 2005	0.122	0.038	0.206	0.004
Herron 2006 (15)	0.123	0.040	0.206	0.004
Sacks 1984	0.130	0.044	0.216	0.003
Harman 2008	0.130	0.045	0.214	0.003
Greene 2006 (13)	0.131	0.046	0.217	0.003
Ballesteros 2004 (2)	0.134	0.045	0.222	0.003
Ginsberg 1994 (9)	0.135	0.049	0.222	0.002
Herron 2006 (14)	0.136	0.047	0.224	0.003
Ginsberg 1994 (8)	0.136	0.049	0.222	0.002
Ginsberg 1994 (7)	0.136	0.049	0.223	0.002
Mutungi 2008	0.137	0.052	0.223	0.002
Baumgartner 2013 (3)	0.138	0.050	0.225	0.002
Baumgartner 2013 (4)	0.138	0.052	0.224	0.002
Ginsberg 1995 (11)	0.140	0.052	0.229	0.002
Herron 2006 (17)	0.144	0.057	0.231	0.001
Ginsberg 1995 (10)	0.144	0.056	0.232	0.001
Missimer 2017	0.144	0.056	0.233	0.001
Chakrabarty 2004 (5)	0.144	0.059	0.230	0.001
Greene 2006 (12)	0.146	0.062	0.230	0.001
Herron 2006 (16)	0.150	0.063	0.236	0.001
Ballesteros 2004 (1)	0.150	0.064	0.236	0.001
	0.136	0.052	0.219	0.001

Difference in means (95% CI) with study removed

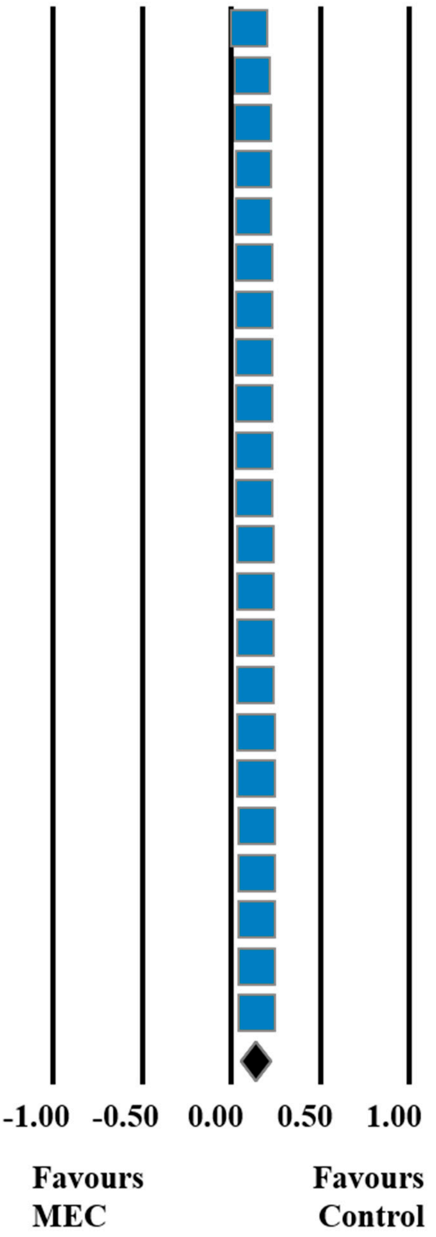

Figure S2
Small study effects in LDL/HDL ratio

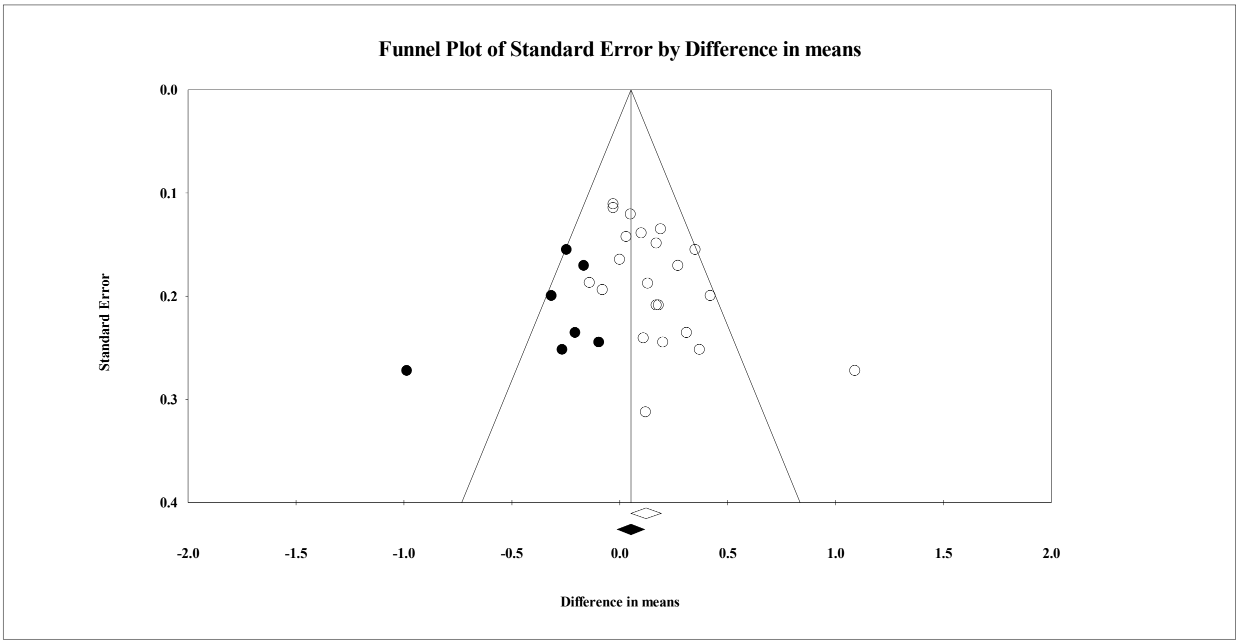

Egger's regression intercept

Intercept	2.13767
Standard error	0.78999
95% lower limit (2-tailed)	0.48978
95% upper limit (2-tailed)	3.78557
t-value	2.70594
df	20.00000
P-value (1-tailed)	0.00680
P-value (2-tailed)	0.01360

Duval and Tweedie's trim and fill

		**Fixed Effects**			**Random Effects**			**Q Value**
	Studies Trimmed	Point Estimate	Lower Limit	Upper Limit	Point Estimate	Lower Limit	Upper Limit	
**Observed values**		0.12205	0.05221	0.19188	0.13563	0.05239	0.21887	28.05723
**Adjusted values**	7	0.05175	-0.01174	0.11525	0.05583	-0.03972	0.15139	58.42167

Figure S3

Sensitivity analysis of LDL-C

Study name	Statistics with study removed: Point	Lower limit	Upper limit	p-Value
Chakrabarty 2004 (5)	7.253	3.928	10.578	0.000
Herron 2006 (14)	7.500	3.906	11.094	0.000
Herron 2006 (17)	7.562	3.890	11.235	0.000
Ballesteros 2004 (2)	7.711	3.885	11.538	0.000
Greene 2006 (12)	7.760	4.108	11.413	0.000
Katz 2005	7.894	3.996	11.792	0.000
Mutungi 2008	7.978	4.233	11.723	0.000
Ginsberg 1995 (11)	8.010	4.116	11.903	0.000
Baumgartner 2013 (3)	8.030	4.231	11.828	0.000
Sacks 1984	8.106	4.248	11.963	0.000
Ginsberg 1994 (8)	8.108	4.259	11.957	0.000
Baumgartner 2013 (4)	8.195	4.367	12.022	0.000
Ginsberg 1994 (7)	8.267	4.386	12.147	0.000
Ginsberg 1994 (9)	8.279	4.405	12.153	0.000
Missimer 2017	8.283	4.275	12.291	0.000
Rueda 2013	8.414	4.537	12.292	0.000
Ginsberg 1995 (10)	8.454	4.564	12.345	0.000
Chakrabarty 2004 (6)	8.571	4.847	12.295	0.000
Harman 2008	8.577	4.767	12.386	0.000
Greene 2006 (13)	8.593	4.883	12.304	0.000
Herron 2006 (16)	8.631	4.863	12.399	0.000
Ballesteros 2004 (1)	8.701	4.825	12.577	0.000
Herron 2006 (15)	8.776	5.013	12.539	0.000
	8.150	4.447	11.853	0.000

Difference in means (95% CI) with study removed

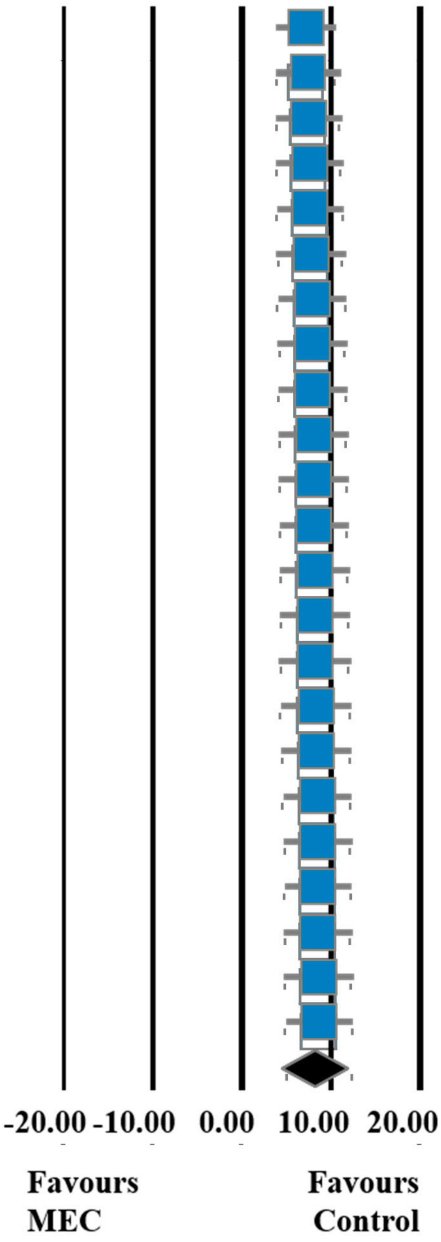

Figure S4

Small study effects in LDL-C

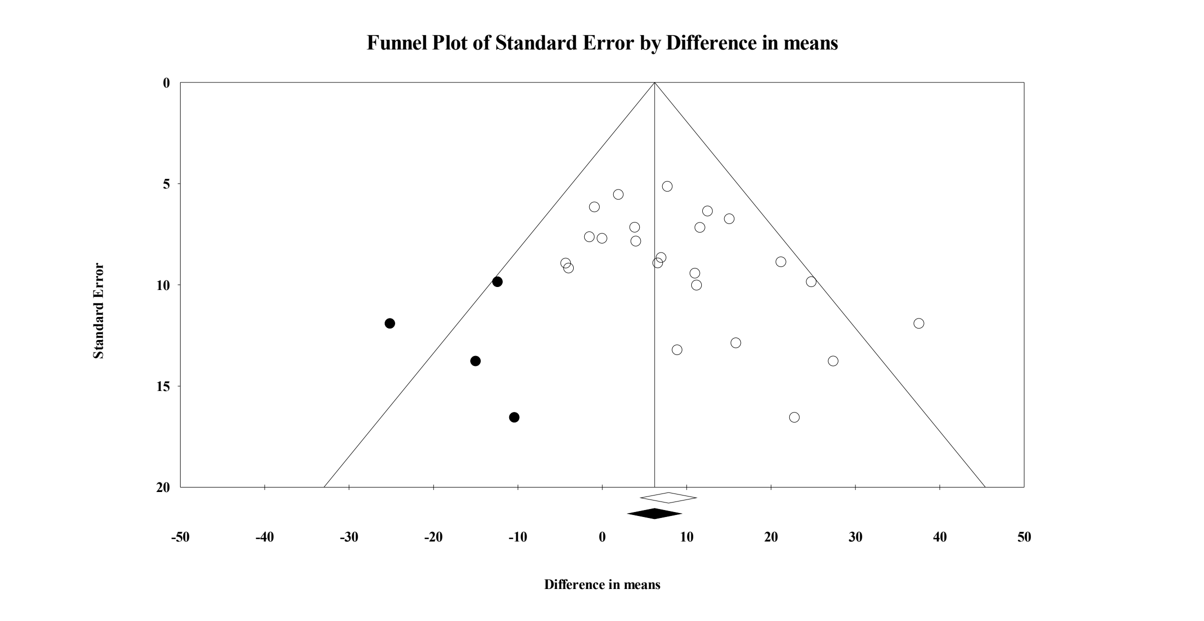

Egger's regression intercept

Intercept	1.78204
Standard error	0.76914
95% lower limit (2-tailed)	0.18253
95% upper limit (2-tailed)	3.38154
t-value	2.31694
df	21.00000
P-value (1-tailed)	0.01535
P-value (2-tailed)	0.03069

Duval and Tweedie's trim and fill

		Fixed Effects			Random Effects			Q Value
	Studies Trimmed	Point Estimate	Lower Limit	Upper Limit	Point Estimate	Lower Limit	Upper Limit	
**Observed values**		7.85470	4.56287	11.14652	8.15019	4.44732	11.85306	26.60672
**Adjusted values**	4	6.20659	3.03026	9.38292	6.31583	2.13124	10.50041	41.41692

Figure S5
Sensitivity analysis of HDL-C

Study name	Statistics with study removed			
	Point	Lower limit	Upper limit	p-Value
Herron 2006 (14)	0.978	-0.611	2.567	0.228
Mutungi 2008	1.008	-0.575	2.590	0.212
Greene 2006 (13)	1.061	-0.525	2.647	0.190
Herron 2006 (17)	1.072	-0.548	2.692	0.195
Ballesteros 2004 (2)	1.080	-0.570	2.730	0.200
Missimer 2017	1.103	-0.493	2.700	0.176
Ballesteros 2004 (1)	1.122	-0.458	2.703	0.164
Ginsberg 1995 (11)	1.152	-0.428	2.733	0.153
Baumgartner 2013 (4)	1.159	-0.411	2.729	0.148
Baumgartner 2013 (3)	1.175	-0.404	2.754	0.145
Rueda 2013	1.184	-0.403	2.771	0.144
Ginsberg 1995 (10)	1.202	-0.377	2.782	0.136
Ginsberg 1994 (8)	1.211	-0.388	2.810	0.138
Greene 2006 (12)	1.215	-0.377	2.808	0.135
Herron 2006 (15)	1.217	-0.388	2.822	0.137
Sacks 1984	1.286	-0.288	2.860	0.109
Ginsberg 1994 (7)	1.287	-0.315	2.888	0.115
Chakrabarty 2004 (5)	1.291	-0.311	2.894	0.114
Ginsberg 1994 (9)	1.296	-0.303	2.895	0.112
Chakrabarty 2004 (6)	1.381	-0.197	2.958	0.086
Herron 2006 (16)	1.413	-0.228	3.053	0.092
Katz 2005	1.430	-0.182	3.041	0.082
Harman 2008	1.536	-0.064	3.137	0.060
	1.211	-0.350	2.771	0.128

Difference in means (95% CI) with study removed

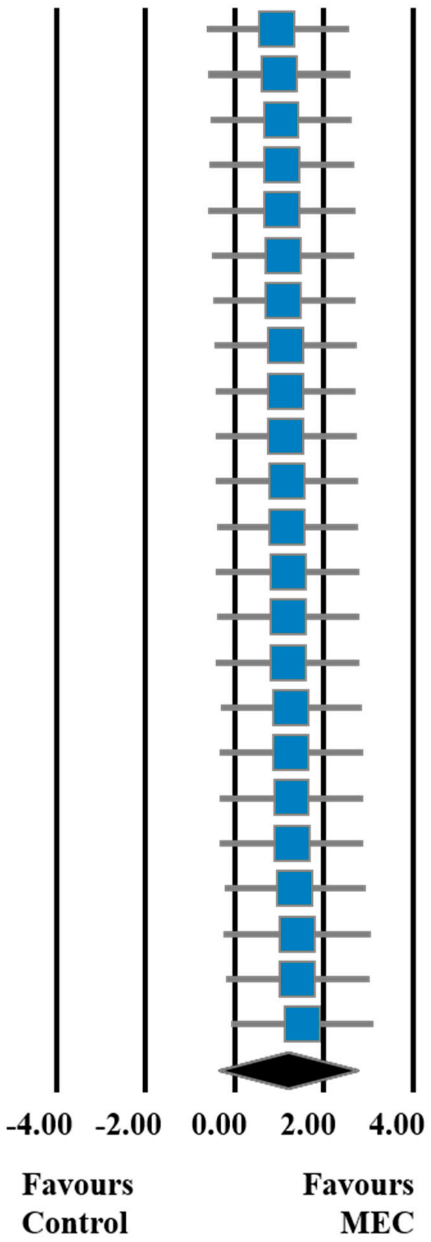

Figure S6
Small study effects in HDL-C

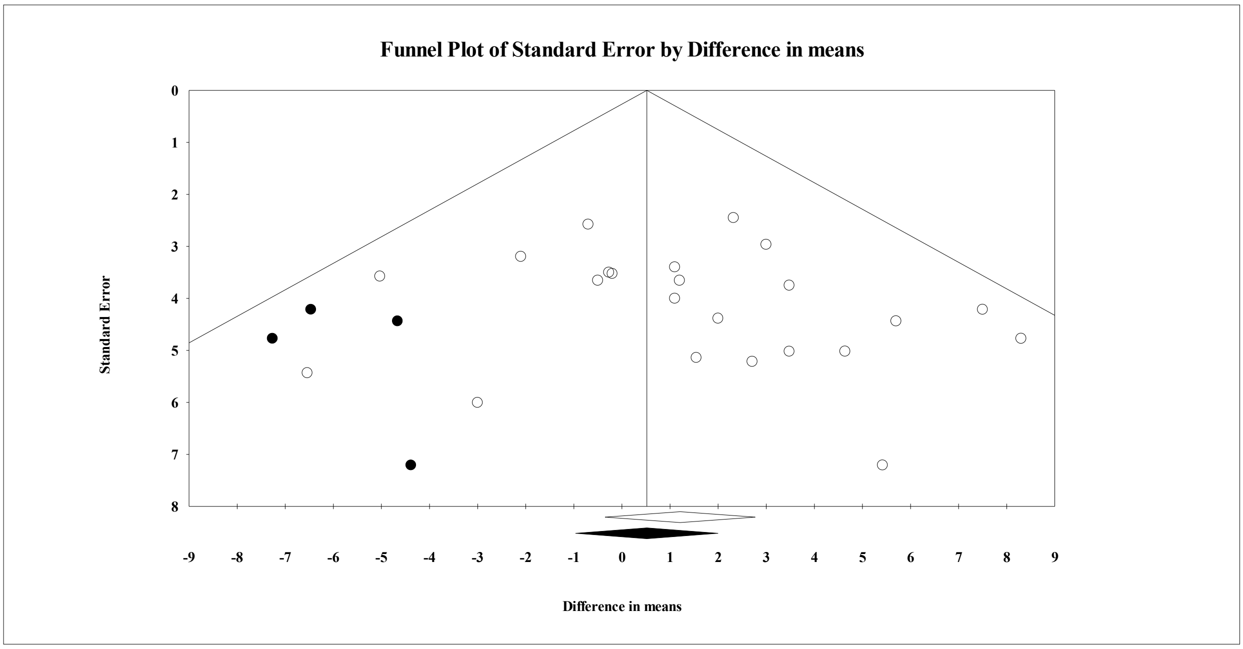

Egger's regression intercept

Intercept	0.61440
Standard error	0.69262
95% lower limit (2-tailed)	-0.82597
95% upper limit (2-tailed)	2.05478
t-value	0.88708
df	21.00000
P-value (1-tailed)	0.19254
P-value (2-tailed)	0.38508
